# Do Large Ears Have an Advantage in Preventing Airborne Transmission?

**DOI:** 10.7759/cureus.30335

**Published:** 2022-10-15

**Authors:** Komiya Kosaku, Kazufumi Hiramatsu, Jun-ichi Kadota

**Affiliations:** 1 Respiratory Medicine and Infectious Diseases, Oita University Faculty of Medicine, Yufu, JPN

**Keywords:** the number of masks, more masks, airborne transmission, infection control, covid-19, leakage, large ear, mask

## Abstract

Although double masking provides better protection against COVID-19 than single masking, the exact number of masks needed to achieve the most significant protection has not been determined to date. The present study investigated the changes in leakage with the use of surgical masks in a healthy person in terms of the number of masks. When the number of masks reached the upper limit that could be worn on the ears, large plastic ear attachments were used for each ear. The leakage rate related to the use of a single mask was approximately 50%, and with two masks, it was significantly reduced to approximately 30%. However, the leakage rate increased to approximately 45% with the use of three and four masks. When more than four masks were worn, the rate gradually decreased. Conversely, the rate for three-seven masks was not significantly lower than that for two masks. Although individuals with large ears might be able to wear more than five masks, the use of more than two masks would not be significantly better than the use of two masks.

## Introduction

During the COVID-19 pandemic, the use of masks was widely recommended to prevent the transmission of severe acute respiratory syndrome coronavirus 2. Wearing masks substantially protects exhaled respiratory droplets and aerosols from wearers with infections as well as reduces the exposure of wearers without infections to these particles [1,2]. On February 10, 2021, the Centers for Disease Control and Prevention announced that the use of two face masks or double masking can more effectively reduce a person’s exposure to coronavirus particles compared with a single mask [3]. However, it has not been determined how many masks are needed to achieve the most significant protective effects. Notably, individuals with large ears tend to wear more masks than those with small ears. If wearing more masks provides significant beneficial effects, people with large ears would have the advantage of preventing airborne transmission. Therefore, this study aimed to investigate the effectiveness of wearing more masks using large ear attachments.

## Case presentation

The first author investigated the changes in leakage from surgical masks by the number of masks. The same brand of surgical masks (BLISSMASK®, Blissmedical, Fukuoka, Japan) was used, and the leakage rate was assessed by a mask fitting tester (MT-05U Mask Fitting Tester, SHIBATA, Saitama, Japan) in accordance with the operating instructions (https://www.sibata.co.jp/wpcms/wp-content/themes/sibata/en/pdf/090200-08_Mask_Fitting_Tester_MT-05U.pdf). The tester performance was approved in accordance with the Japanese Industrial Standards (JIS) as JIS T8150, which are coordinated by the Japanese Industrial Standards Committee (https://www.sibata.co.jp/item/8269/). This tester measures the number of particles in the room (outside the mask) and inside the mask alternately using a test guide bar to quantify how tightly the mask fits the face. After wearing a certain number of masks, the test guide bar (non-destructive mask test) was directly inserted into the mask on the mouths, and the measurement of particles was performed. We started testing with a single mask and added masks to it one by one. When the number of masks reaches the maximum limit to be worn on the ears, the first author wore large plastic ear attachments (Tsukemimi Jambo®, CLEARSTONE Co., Ltd., Tokyo, Japan) on each ear. The measurements were repeated five times by the author and the leakage rates according to the number of masks worn were compared with the ANOVA test and Tukey’s test as a post-hoc test using the Statistical Package for the Social Sciences software version 22 (IBM Japan, Tokyo, Japan). For two-tailed analyses, 95% confidence intervals were calculated.

**Figure 1:**
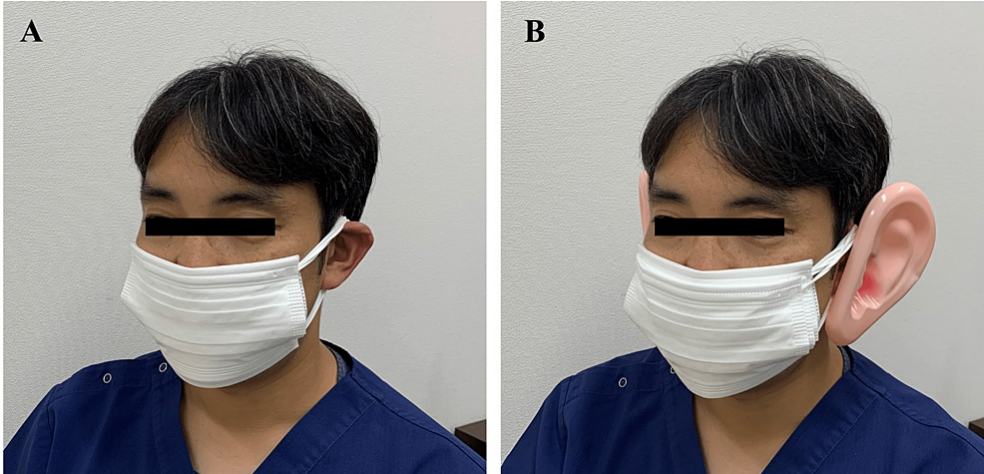
Pictures of the first author wearing five masks (A) and seven masks with large ear attachments (B).

As a result, the author was able to wear up to five pieces of masks (Figure 1A), and when the sixth mask was worn, the ears were congested, and all masks were torn off. After wearing the large ear attachments, which make more mask loops hooked on the ear, the masks were eventually worn up to seven pieces (Figure 1B).

**Figure 2:**
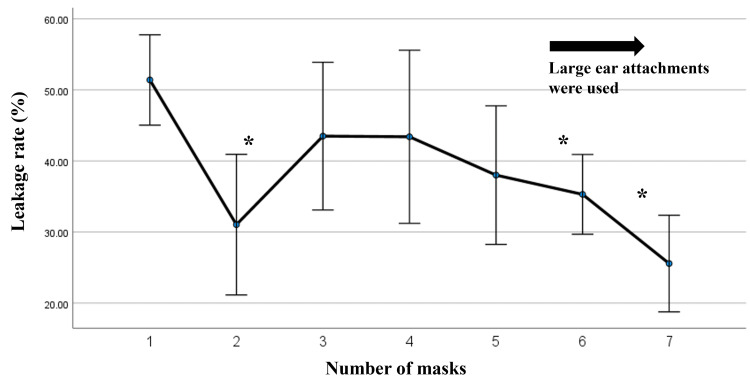
Leakage rate from masks by the number of masks worn. The measurements were repeated five times and the results were shown as average with a 95% confidence interval. The leakage rates in two, six, and seven masks were significantly lower than in a single mask based on the results of the ANOVA test and Tukey’s test as a post-hoc test (*).

The leakage rate for wearing a single mask was approximately 50%, with two masks significantly reducing the rate to around 30% (Figure 2).

The leakage rate increased to approximately 45% when wearing three and four masks and declined gradually when wearing five, six, and seven masks. The leakage rates for six and seven masks were significantly lower than a single mask based on the results of the ANOVA and Tukey’s tests as a post-hoc test. However, the use of six and seven masks was not significantly better than wearing two masks.

## Discussion

This investigation confirmed that two masks may have beneficial effects in preventing airborne transmission compared with a single mask. However, wearing three to five masks did not add favorable effects probably because thickened masks may lead to mask-side leakage. The large ear attachments do not directly affect the tension of the elastic string or fitness when wearing multiple masks. Thus, if less than six masks are worn with large ear attachments, the effects would be identical to that without large ear attachments. Interestingly, wearing six and seven masks with large ear attachments significantly decreased the leakage compared with single masking. Moreover, wearing too many masks adds tension to the strings to which the mask is tightly adhered, which might explain the lower leakage rate. Ear size in adults is known to become bigger as people age, and it is probably associated with changes in collagen [4,5]. Elderly people might be able to wear more than five masks, but we would not recommend too many masks because it was not found to have superiority over double masking. Additionally, wearing too many masks makes the ears get congested, which could lead to difficulties in respiration and speaking. However, this study has several limitations. The leakage rate is a surrogate marker of risk for airborne transmission, and the results may not directly link to the actual transmission risk. Furthermore, the data was obtained from the first author as a case report. Hence, a large-scale study might be needed to confirm the findings.

The decision to wear masks is clearly influenced by certain factors including public health recommendations and government mandates, race and cultural norms, geography, household income, age, and personal attitudes [6]. The custom of wearing masks has been encouraged in East Asia, especially in Japan during the COVID-19 pandemic, even in situations with no infection risk. A nationwide survey in Japan revealed that people are willing to conform to societal norms with regard to wearing masks and feel relief from the anxiety of contracting a viral illness [7]. These social psychological motivations may explain the high rate of masking in Japan, and elderly people tend to be susceptible to the norm. However, wearing more than two masks should not be recommended even in people with large ears.

## Conclusions

People with large ears may be able to wear more masks than those without. Although wearing more than five masks seems more beneficial than using a single mask, their effectiveness would not be significantly better than double masking. Therefore, wearing more than two masks would provide no further protection, and large ears do not appear to have any benefit in preventing airborne transmission.
